# Supplementary material for: Efficacy and Safety of COVID-19 Vaccines in Phase III Trials: A Meta-Analysis
Source: Vaccines (Basel). 2021 Jun 1;9(6):582. doi: 10.3390/vaccines9060582 (PMC8228087; doi:10.3390/vaccines9060582)
Supplement: Supplementary file 1 [file vaccines-09-00582-s001.zip › vaccines-1244876-supplementary.pdf]

Efficacy and Safety of COVID-19 Vaccines in Phase III Trials: A Meta-Analysis

Haoyue Cheng [1,2], Zhicheng Peng [1,2], Wenliang Luo [1,2], Shuting Si [1,2], Minjia Mo [1,2], Haibo Zhou [1,2], Xing Xin[1,2], Hui Liu [3], Yunxian Yu [1,2,*]

1. Department of Public Health and Department of Anesthesiology, the Second Affiliated Hospital of Zhejiang University School of Medicine, Hangzhou, China
2. Department of Epidemiology & Health Statistics, School of Public Health, School of Medicine, Zhejiang University, Hangzhou, China
3. Sir Run Run Shaw Hospital, School of Medicine, Zhejiang University

* Correspondence: yunxianyu@zju.edu.cn

Table S1. Literature search terms used for PubMed. The final search strategy applied to conduct this meta-analysis is reported at step #21.

#	**Search strategy**
1	corona virus OR coronavirus
2	coronavirinae
3	coronaviridae
4	betacoronavirus OR beta coronavirus
5	COVID19 OR COVID 19 OR COVID2019 OR COVID 2019
6	nCoV
7	"CoV 2" OR CoV2
8	2019nCoV OR 2019 nCoV
9	severe acute respiratory syndrome coronavirus 2
10	SARS CoV 2 OR sarscov2
11	#1 OR #2 OR #3 OR #4 OR #5 OR #6 OR #7 OR #8 OR #9 OR #10
12	vaccin*[tiab]
13	immuniz*[tiab]
14	inocul*[tiab]
15	#12 OR #13 OR #14
16	#11 AND #15 (12345)
17	"COVID19 vaccin*" OR “anti-sars-cov-2 agent'” OR anti-sarscov2 agent OR “sars-cov-2 vaccine”
18	#16 OR #17 (13128)
19	Humans[Mesh] OR human*[tiab] OR volunteer*[tiab] OR participant*[tiab] OR subject*[tiab] OR people[tiab]
20	(clinical[tiab] AND trial[tiab]) OR clinical trials as topic[Mesh] OR clinical trial[pt] OR random*[tiab] OR “random allocation”[Mesh]
21	#18 AND #19 AND #20 (806)

Table S2. Literature search terms used for the Cochrane Library. The final search strategy applied to conduct this meta-analysis is reported at step #23.

#	**Search strategy**
1	MeSH descriptor: [Coronavirus] explode all trees
2	MeSH descriptor: [Coronavirus Infections] explode all trees
3	MeSH descriptor: [Coronaviridae] explode all trees
4	MeSH descriptor: [Betacoronavirus] explode all trees
5	coronavir* OR corona virus OR betacoronavir* OR beta coronavirus
6	MeSH descriptor: [COVID-19] explode all trees
7	COVID19 OR COVID 19 OR COVID 2019
8	nCoV OR CoV 2 OR CoV2 OR 2019 nCoV
9	MeSH descriptor: [SARS-CoV-2] explode all trees
10	severe acute respiratory syndrome coronavirus 2 OR SARS CoV 2 OR sarscov2
11	#1 OR #2 OR #3 OR #4 OR #5 OR #6 OR #7 OR #8 OR #9 OR #10
12	MeSH descriptor: [Vaccines] explode all trees
13	MeSH descriptor: [Immunization] explode all trees
14	(vaccin*):ti,ab,kw OR (immuniz*):ti,ab,kw OR (inocul*):ti,ab,kw
15	#12 OR #13 OR #14
16	#11 AND #15 (481)
17	MeSH descriptor: [COVID-19 Vaccines] explode all trees
18	COVID19 Vaccin* OR COVID 19 Vaccin* OR anti sars cov 2 agent OR anti sarscov2 agent OR sars cov 2 vaccin* OR sarscov2 vaccin*
19	#17 OR #18
20	#16 OR #19 (532)
21	(human*):ti,ab,kw OR (volunteer*):ti,ab,kw OR (participant*):ti,ab,kw OR (subject *):ti,ab,kw OR (people*):ti,ab,kw
22	("clinical trial"):ti,ab,kw OR (random*):ti,ab,kw OR (clinical trial):ti,ab,kw
23	#20 AND #21 AND #22 (301)
24	trial: 277

Table S3. Literature search terms used for Embass. The final search strategy applied to conduct this meta-analysis is reported at step #19.

#	**Search strategy**
1	coronavirus OR "corona virus"
2	Coronavirinae'/exp OR Coronavirinae
3	Coronaviridae'/exp OR Coronaviridae
4	Betacoronavirus'/exp OR 'Betacoronavirus' OR 'beta coronavirus'
5	coronavirus disease 2019'/exp OR 'coronavirus disease 2019' OR 'COVID-19' OR COVID19 OR sarscov2 OR 'COVID-2019' OR COVID2019
6	Severe acute respiratory syndrome coronavirus 2'/exp OR 'Severe acute respiratory syndrome coronavirus 2' OR 'nCoV' OR 'SARS-CoV-2'
7	"CoV 2" OR CoV2
8	"2019-nCoV" OR 2019nCoV
9	#1 OR #2 OR #3 OR #4 OR #5 OR #6 OR #7 OR #8
10	vaccine'/exp OR vaccin*:ti,ab,kw
11	immunization'/exp OR immuniz*:ti,ab,kw
12	inocul*:ti,ab,kw
13	#10 OR #11 OR #12
14	#9 AND #13 (14701)
15	anti-sars-cov-2 agent'/exp OR 'anti-sars-cov-2 agent' OR 'sars-cov-2 vaccine'/exp OR 'sars-cov-2 vaccine' OR "covid19 vaccin*" OR "covid 19 vaccin*"
16	#14 OR #15 (15952)
17	human'/exp OR human:ti,ab,kw OR volunteer*:ti,ab,kw OR participant*:ti,ab,kw OR subject*:ti,ab,kw OR people:ti,ab,kw
18	clinical trial (topic)'/exp OR 'randomization'/exp OR randomization:ti,ab,kw OR (clinical trial):ti,ab,kw OR random*:ti,ab,kw
19	#16 AND #17 AND #18 (1355)

Table S4. Literature search terms used for Medrxiv, Wanfang and CNKI.

Database	Search strategy
Medrxiv	COVID-19 AND vaccin* AND "clinical trials" (4920)
Wanfang	(主题:(新型冠状病毒) OR 主题:(新冠病毒) OR 主题:(新型冠状病毒肺炎) OR 主题:(新冠肺炎)) AND 主题:(疫苗) AND (主题:(临床试验) OR 主题:(随机)) (同义词扩展) (102)
CNKI	TKA=('新型冠状病毒'+'新冠病毒'+'新型冠状病毒肺炎'+'新冠肺炎') and TKA=('疫苗') and TKA=('临床试验+随机') (同义词扩展) (94)

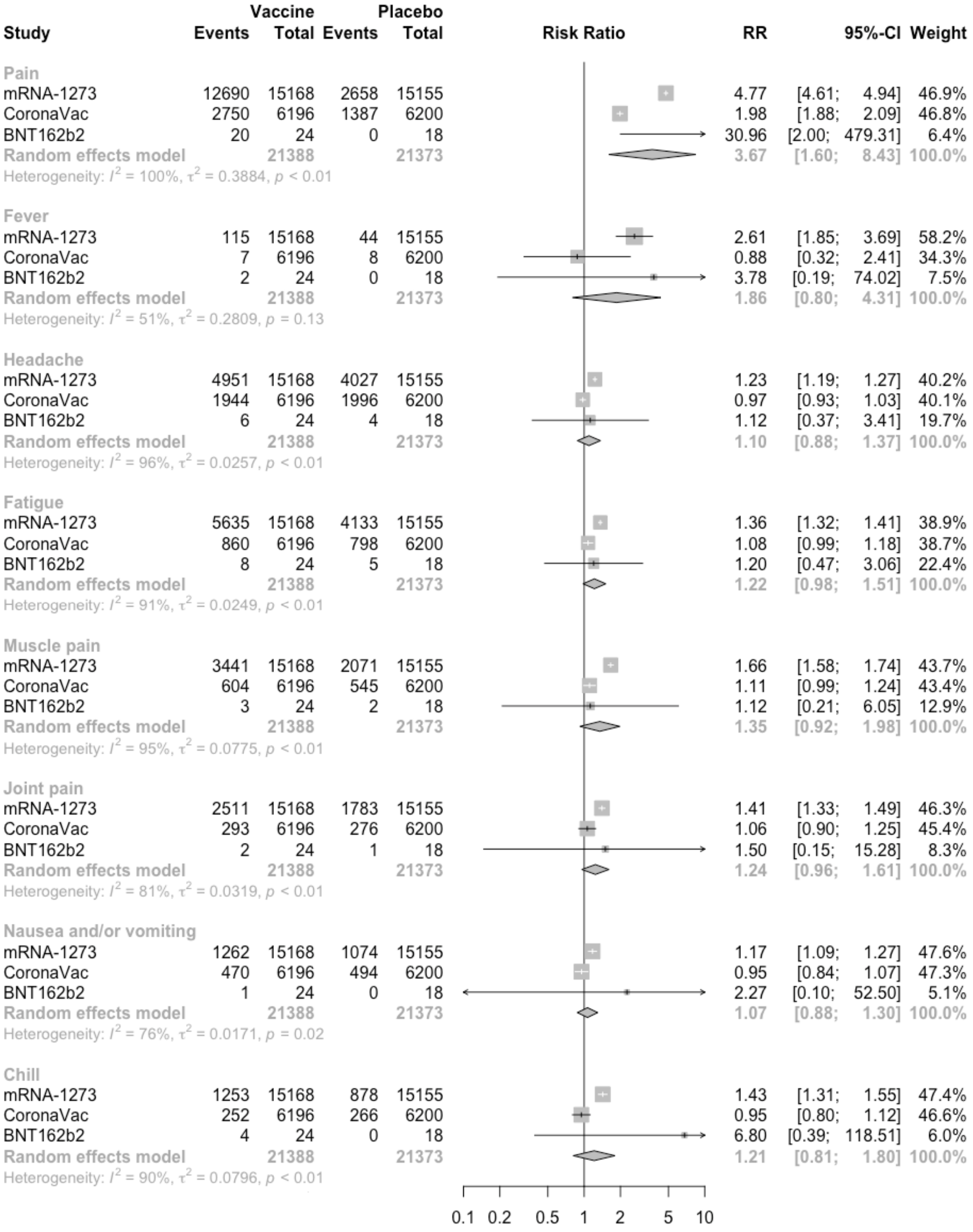

Figure S1. Meta-analysis of effect of vaccine on specific adverse events between vaccine and placebo groups after dose 1

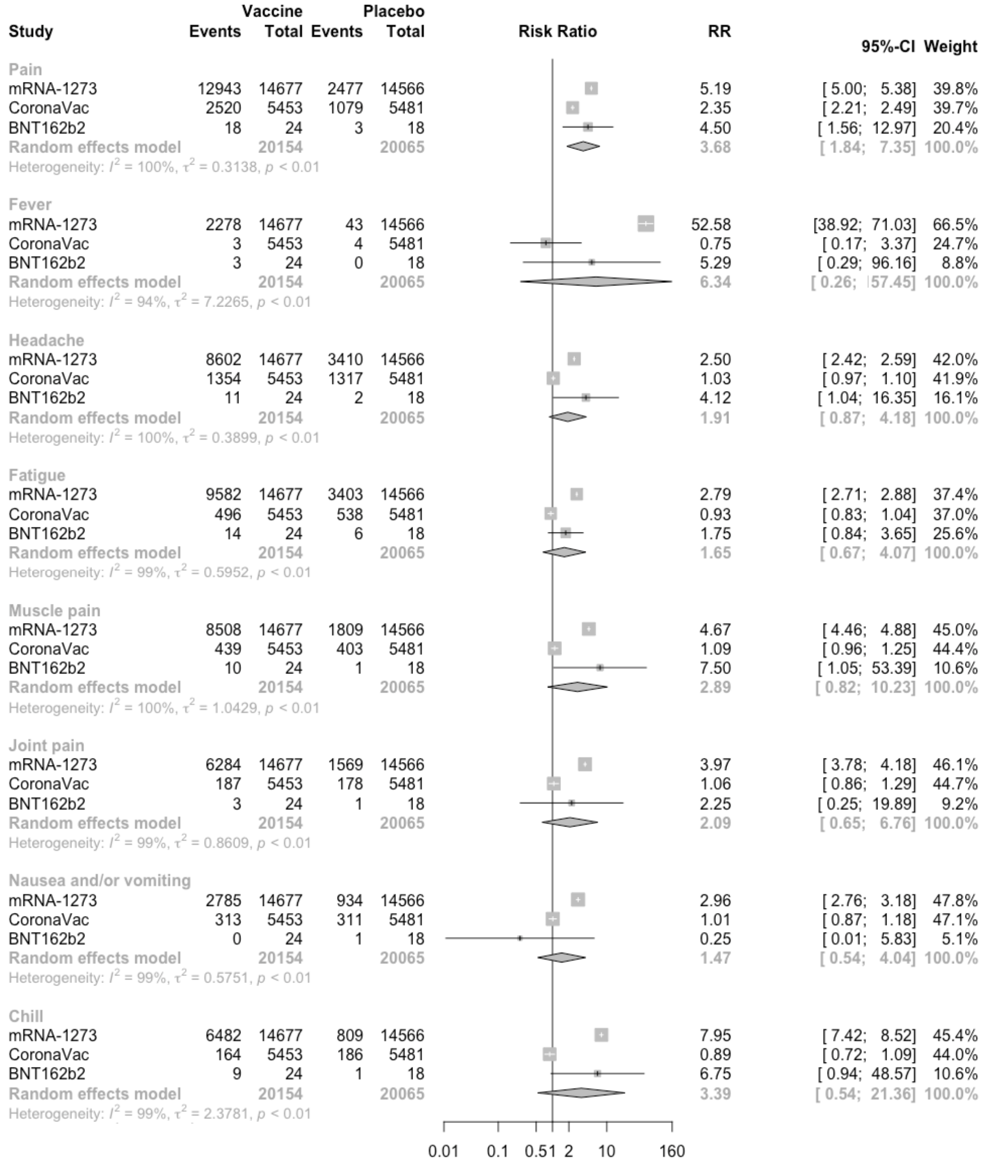

Figure S2. Meta-analysis of effect of vaccine on specific adverse events between vaccine and placebo groups after dose 2
